# Digital business, work and consumption in a service relation: a case study of a delivery platform company

**DOI:** 10.3389/fsoc.2024.1454305

**Published:** 2025-01-02

**Authors:** Sofia Alexandra Cruz, Catarina Sales Oliveira

**Affiliations:** ^1^Faculty of Economics, University of Porto, Porto, Portugal; ^2^Institute of Sociology, University of Porto, Porto, Portugal; ^3^Faculty of Social and Human Sciences, University of Beira Interior, Covilhã, Portugal; ^4^Centre for Research and Studies in Sociology, CIES - ISCTE, University Institute of Lisbon, Lisbon, Portugal

**Keywords:** platform economy, digital business, work, consumption, service relation, delivery platform

## Abstract

The platform economy has contributed to new ways of organising business, work, and consumption. To understand the shape and scope of these changes, it is crucial to pay simultaneous attention to these three domains. The new ways of organising, dividing and coordinating work are interlinked with specific ways of consuming services made available by digital platforms. This article analyses a case study of a delivery platform company with a specific business model marked by strong territorial roots and promoting relational proximity between the platform, workers, and clients. It proposes an approach to the service relation between the platform, workers, and clients allowing to explore institutional, intersubjective and spatial–temporal dimensions that shape it. Intersubjective, because they point to particular modes of communication and involvement between the parties. Institutional, as the platform reflects the organisational environments within which it operates. Spatio-temporal, as this service relation promotes flexible spatial and temporal arrangement that articulates various urban actors.

## 1 Introduction

Digital work platforms have changed the way work is organised (Del Bono, 2019; Pesole et al., 2018), thus changing traditional working relationships (Graham et al., 2020; Sasikumar and Sersia, 2020; Vallas and Schor, 2020). The main characteristic is that work is performed through a digital device without the organisational layout of a company (Da Silva, 2020), which traditionally consists of two types of structures. On the one hand, a technical-functional structure, in which workers are assigned to different workplaces according to their skills, thus performing different roles that complement each other in the pursuit of a common goal. On the other hand, a social structure in which workers establish horizontal relationships with their co-workers and vertical relationships with their hierarchical superiors, the latter being responsible for delegating tasks. In return, in work carried out through digital platforms, the mandatory use of technological devices breaks with the traditional model of a company, in the sense that everything is done in a virtual environment. In other words, there is no face-to-face relationship between worker and client, the work is coordinated, regulated and monitored through algorithms, and in this way the role of delegating tasks by hierarchical superiors is replaced, and the work is disseminated to a crowd, thus replacing the functional structure of a company, and often without the worker being aware of the final goal of their work. Furthermore, workers on these platforms are hired as casual labour, sometimes paid by the piece, which is a return to the work model of the old days (ILO, 2018, 2021).

Whilst work has been deeply analysed in scientific research on digital platforms, consumption has been considerably less problematised (Cruz and Gameiro, 2023; Alonso and Fernández Rodríguez, 2021). However, the relationship between work and consumption in the platform economy cannot be overlooked, as they are two dimensions of the same reality. Based on a case study about a Portuguese company, this article aims to discuss this relationship by analysing the triangular service relation between platforms, workers and clients. This case study presents a delivery platform company with a specific business model characterised by a territorial rootedness that promotes relational proximity between platform, workers and clients. The research seeks to operationalise an analytical proposal on the triangular service relation between platforms, workers and clients (Cruz and Gameiro, 2023), to specifically discuss its institutional, intersubjective and socio-spatial characteristics.

Concerning the institutional dimensions, it is important to problematise the structure and dynamics of the business operation of these digital platforms. They invite workers and clients to standardise a set of routines for accessing work and consumption, and to relate to each other through mostly virtual, but also face-to-face contact, especially in the case of the labour universe of deliveries (ILO, 2024). On the other hand, these institutional dimensions suggest taking into account the fact that they are shaped by the institutional contexts in which they operate (Vallas and Schor, 2020), with different economic, political and legal characteristics. The intersubjective dimensions point to the importance of taking into account the specificities of communication dynamics, encouraging reflection on the diversity of cultural and linguistic devices that take place in a context of virtual communication through digital platforms (Aroles et al., 2019). The spatio-temporal dimensions highlight the fact that workers are very often hired globally, so that clients have access to the greatest possible diversity of potential workforce available, in a web of social relations guided by particular preferences or by the inexistence of alternatives. In this context, it is worth stressing the prominence of migrant workers absorbed by digital platforms (van Doorn and Vijay, 2021), especially delivery platforms, through which these workers aspire to eventual legalisation in their host countries.

After explaining the aim and theoretical framework of the article, the next section presents its methodological approach, followed by a description of the data collected and, finally, a discussion and concluding remarks.

## 2 Method

In this section we start by presenting the company and the region. Next, we discuss the methodological approach, providing details about our options and the data collection procedures.

This research is a case study of a young delivery company with some unique characteristics: being a pioneering delivery company in a deprived region of the Portuguese countryside; featuring an entrepreneurial initiative of young university graduates and claiming to have social concerns and to provide a highly personalised client interaction. These characteristics caught the attention of the researchers, who were interested in the phenomenon of delivery specially from the consumption perspective.

Xicos was founded in 2018 in Covilhã. Since then it has grown to two other nearby cities with the same business model, Fundão and Castelo Branco, and since 2021, it has been expanding through franchising to 12 more municipalities, all in the centre of the country, except Elvas, in the Alentejo Region. In this study, we analysed Xicos’ head office in Covilhã.

Covilhã is located in Cova da Beira, a small region in the mountainous countryside of Portugal. Orlando Ribeiro describes it as a “depression between the high hills of Serra da Estrela and Serra da Gardunha that close it from North, South and West” (Ribeiro, 1949, p. 24). It has an area of only 1,373 km2 and consists of three municipalities: Belmonte, Covilhã and Fundão. Covilhã is the biggest, with 46,455 inhabitants, followed by Fundão with 26,503 and Belmonte with just 6,204. Covilhã hosts a university that is responsible for creating jobs in the region and also for dynamizing people’s mobilities and migrations, which since 2018 has become more expressive. Fundão has also been growing due to municipal policies attracting population and companies. However, it is essential to highlight that the most critical demographic dynamic of the region is the population decline, mainly youngsters, due to internal migration to the coast and emigration.

When the company was founded in 2018, no delivery companies existed in the Cova da Beira region. In Fundão, Xicos is still the only delivery company. At a national level, currently “Uber Eats is the main Online Food Delivery player in Portugal (with a market share of 30%)” (Loureiro, 2021, p.12), but in the centre of the country, local food delivery companies play a significant role as there are several cities that the more prominent delivery companies do not yet cover.

Xicos presents itself as a brand of more than delivery. It claims to be a community, highlighting its Portuguese heritage, as we can see in the way they define their mission:

"Xicos are not just a brand: they are not Chicos, moody or grumpy guys, they are not just couriers and they are not just about food. They are a 100% Portuguese brand; they are an app and a website; they are patriotic, clever and funny; they are people; they are a community; they are purple and orange and they have more attitude than a Portuguese *feijoada*^1^." (Xicos' website)

In 2023, the company reached a turnover of one million euros. In 2021, there was a significant change in how the company operated and its structure with the merger with Upeats. This 100% Portuguese start-up began operating in 2020 in Greater Lisbon. This merger enabled a major technological leap forward. It also involved a change in the company’s image and marketing. In terms of employees, Xicos started with 2 people and currently has a team of 154.

Concerning the methodological options of the research, it is a case study identified as relevant due to the particularities described above. The aim was to gain an in-depth and comprehensive understanding of the company’s structure and operation and its relational dynamics in the region, namely with the clients. Therefore, we conducted semi-directive interviews with nine people, in particular one founder, four employees and four clients, between June 2023 and January 2024. The founder is a former student at the University of Beira Interior, where one of the authors is a professor. This condition was important for carrying out the empirical research, as the founder was willing to be interviewed.

As we were at the company’s headquarters for 1 day, we chose to approach one worker directly and, following a snowball strategy, we managed to contact three other workers. With regard to clients, one of the authors had privileged contact with some of the company’s clients, so we interviewed those who were available. The interview with the founder focused on the themes of ownership and business dynamics; work organisation model, work and consumption, data extraction function and platform politics. The interviews with workers were oriented around reasons for seeking work at Xicos, working hours, satisfaction and motivation in the field, type of relationship with customers and future work perspectives. The customer interviews focused on the reasons for being a Xicos customer, comparative analysis between Xicos and other delivery platforms, the type of communication the company establishes with the customer, and the company’s role in the local community.

The interviews took place in the headquarters of the company (workers), and by *zoom* (clients), between June 2023 and January 2024. The respondents signed an informed consent prepared by the researchers, authorising the use of data for research purposes.

At the same time, we gathered information on the case as much as possible by collecting previous studies, newspaper articles and interviews in the media and by systematically monitoring the institutional communication available on the Xicos website. These informations helped with the analytical framing of the interviews.

## 3 Results

This section is structured according with the analytical model used in the research (Cruz and Gameiro, 2023) and explained above. We provide data for each analytical dimension studied - institutional, problematising the structure and dynamics; intersubjective, point out the importance of the intensity and quality of comunication flows and spatio-temporal, analysing the situational context of the business case.

### 3.1 Institutional dimension

The discussion of this dimension is anchored in Mintzberg (2023)'s perspective on the relationship between organisational structure and the dynamics of its operation. In particular, the basic components, work coordination mechanisms and organisational workflows.

The main basic components of Xicos are the strategic apex and the operational centre. The strategic apex corresponds to the company’s administration, which guarantees the effective implementation of the company’s mission and is occupied by four people, former students, university colleagues and former couriers of the very company they founded.

"At the time, it was a bit of an entrepreneurial motivation, so to speak, apart from anything else (...) but it was also created to fill a gap that existed here in our area, in this case in the city of Covilhã, because there was only home delivery of pizza. (...) It was this ambition. We saw this opportunity, we took it (....) and it worked". (Founding partner, male, 25 years old).

One of the main missions of the company’s strategic apex is to establish strategic partnerships in order to make the company more profitable.

"The logic of the current partnership is one of specialisation. Xicos has been left with logistics and contact with partners and clients: We want the part we're good at. And that's what we've been doing since the beginning. The restaurants, the partnerships, the couriers, everything. (Founding partner, male, 25 years old).

The company’s operational centre is made up of two profiles of professionals: the head office staff and the couriers. The main function of the head office is to centralise communication between clients, restaurants and couriers. At client level, the head office handles phone calls with change requests, as well as service requests from clients who do not place their orders online. In addition to controlling workflows, the head office also assists couriers with delivery issues, streamlining their daily work:

The difference [from the experience of working for this company] is the communication with the head office, they are always ready to help if I have any questions, and today I could not find the number of one of the clients, the house number of this client, I called and then? They said this, this, this and this. They gave me all the support I needed.” (Courier, male, 41 years old).

The fact that the head office workers have already been couriers means that this role acts as a rite of entry into the company (Machado et al., 2013) facilitating workflows and communication, as all recruited workers start in this position.

"I speak in the head office - the head office behind the computer that manages orders and deliveries in the call centre - nobody ever goes there without having been on the ground. You can't speak, and you won't be able to speak, in a correct and heartfelt way if you don't do it". (Founding partner, male, 25 years old)

The head office therefore plays a fundamental role in coordinating all the company’s activities.

With regard to the mechanisms for coordinating work, at the same time that Xicos has adopted a certain standardisation of the work process by no longer having its own fleet of vehicles, the company is still favouring fluid and flexible coordination mechanisms: mutual adjustment and direct supervision. There is flexibility in the organisation of work, with the promotion of tailor-made solutions to problems that arise. This personalised response is possible because there is (still) direct supervision by the founding partners, who are always present, both physically and virtually. The existing WhatsApp group reinforces this connectivity being widely used by the teams. Communication between the head office, the couriers and the restaurants is daily and constant. Internally, informal communication flows are very strong, with mutual support between colleagues and with the supervisors.

"We are synchronised both in terms of the way we work and on a personal level... On the right personal level (...)" (Founding partner, male, 25 years old)

"It's the way (...), (...), treat us, not like bosses, but like friends, because they always help you, with whatever you need" (Courier, male, 41 years old).

In terms of working hours, the couriers’ availability is communicated by them on a weekly basis and adapted to their needs, which seems to contradict the more usual patterns of this job based on place and temporal control practices (Heiland, 2022).

"The couriers' availability to work is shared on a weekly basis, according to people's lives... Sometimes there are people who just want to cover some gaps (...) they work about 6 hours a week" (Founding partner 1, male, 25 years old)

"The couriers can either tell us "give me all the days" or next week "look, I'm sorry, but next week I can only work Monday and Tuesday” (Founding partner, male, 25 years old).

In terms of the relationship with the workers, work control is also flexible. Services are not yet adjusted by algorithms, but manually, and they say that “we do not need to know how many kilometres have been driven this week. No, I do not want to know. What I want to know is (...) how much he’s getting paid” (Founding partner 1, male, 25 years old).

Concerning work, it is important to note that in 2024 Xicos received national media attention because, following an inspection by the Autoridade para as Condições de Trabalho (Labour Conditions Authority) in 2023, a ruling was made recognising the contract of three couriers and the company regularised the situation, being the only company in the country that did not contest the court’s decision (Pereira, 2024).

This flexibility in the organisation of work is also evident in the way the physical workplace in Covilhã is organised and managed:

"Here in Covilhã we have the office (...) it's rented (...) we still have this space, it's big (...) we have to make the best out of it, we have the head office, we have a little room for the couriers, where they can leave things, we have clothes hangers, stools (...) whenever they need to leave things here (...) we're here to find solutions (...) and then we have the office inside." (Founding partner, male, 25 years old).

### 3.2 Intersubjective dimension

This dimension focuses on the forms of external corporate communication (Cornelissen, 2020) practised by Xicos and involving different parties. The logic of support service and advice to clients and partner restaurants is under analysis, as well as the options of coordination with local services.

As mentioned before in relation to the workflow, at Xicos, external communication is an absolutely central part of the business dynamic. Xicos is committed to proximity communication with clients and partner restaurants, providing a telephone line and a contact via WhatsApp for the head office.

"Sometimes we have clients who call the head office and say: 'Hey, what have we got today? (...)' If it's a client of a certain age, we say: 'Well, today we have the special of the day from this restaurant (...). Well, we have Portuguese stew, Valencian rice (...)'" (Founding partner, male, 25 years old).

"It's not just about having the tablet at the partner's place, there are people who have never worked with the delivery system (...) it's necessary to maintain a constant dialogue, a constant helpline, every missed call is a returned call (...) there are people who have never worked with the delivery system, we have partnerships today that if it wasn't for the "hold on, we'll be right there, we'll do a test with you", if we didn't have this, we wouldn't have established the partnerships (...) (Founding partner, male, 25 years old).

Regarding this topic, one client highlighted the different possibilities of virtual and telephone communication offered by Xicos: “we can contact the courier directly, or someone who is always on the helpline, or even via WhatsApp” (Client, female, 41 years old). As one of the company’s first clients, she says that she already knows all the couriers who make the deliveries, which helps to speed up communication with the company, especially in the case of personal deliveries to the workplace or home.

Another client, who consumes products from other platforms similar to Xicos, mentions the advantage of the communication options offered by this company compared to other platform companies:

“There’s an advantage they have that the others do not have, which is that if there’s a problem you can get through to their helpline (...) it’s important to be able to talk to someone, explain what’s going on and why you are not getting your orders.” (Client, female, 39 years old).

Xicos promotes the use of humour in client relationships and as a brand image. The website and the app are full of jokes and there is a section where the expressions of Xicos couriers are published. This approach contributes to the creation of a corporate identity and a differentiated corporate reputation (Urde and Greyser, 2016; Melewar et al., 2012), potentially fostering emotional relationships with clients and within the company. Some messages are strongly present in the speech of the founding partner interviewed, namely being 100% Portuguese - “there is nothing more Portuguese than the name Chico” (Founding partner, male, 25 years old), being local and wanting to promote localism - specifically through partnerships with local restaurants rather than the big globalised chains - “we do not have a partnership with McDonalds” (Founding partner, male, 25 years old), and being socially responsible. Xicos claims to be inclusive by seeking partnerships and offering services that can meet all customer profiles. “We have a target from the restaurant that sells meals for 5 euros [to] (...) people who order lunch and dinner every day.” (Founding partner, male, 25 years old), making some adjustments to the delivery fee to suit the situation and the client profile. The case of blind clients is particularly illustrative in this respect, as Xicos can speed up the ordering process for these clients through its telephone service.

The case of university clients is particularly relevant because, as we mentioned in the previous section of the article, Covilhã is a university city that depends heavily on academic dynamism.

"We always have to be active (...) the question of special offers (...) this week, as we know it's an exam week, we have special offers every day, we did a joint campaign with the students' residence (...) if you buy a pizza, we offer you another one (...). The barbecue menu offers a one and a half litre drink (...) the next day we offer a drink and chips (...) everything is dynamic (...) everything is developed by us, it's not the partner, we have to take the lead (...) (Founding partner, male, 25 years old).

### 3.3 Spatio-temporal dimension

The analysis of this dimension integrates both the discussion on the perspective of platforms in cities, not only as companies, and the perspective of the urban geography of platforms, which defines them as a flexible spatial arrangement with a central role in the organisation of cities (Richardson, 2020). In the case of Xicos, it is a flexible spatial and temporal arrangement that allows the articulation of different urban actors and the exercise of its corporate social responsibility.

The in-depth knowledge of cities and their mobility dynamics, reinforced by the flexibility of the platform, which allows comments to be added, is a differentiating element mentioned by a client: “it’s very simple, we put notes and they come and bring it to us.” (Client, female, 54 years old), as opposed to other competitor companies that rely solely on geolocalisation (address). Another client highlights the option of combining different addresses, such as work and home. If the delivery address is already outside the work area, Xicos suggests alternatives, such as organising an intermediate meeting point with the client.

A client who orders lunch every day during the working week also mentions the possibility of accommodating different addresses and shares that there might be an opportunity to improve the app itself.

“In the settings of the app we have the possibility to change the address, but when we place the order we don't have that possibility and I think this sometimes causes some confusion because we don't remember when we placed the order (...) I think this would be something to improve, to offer the possibility to define the address when we place the order." (Client, female, 41 years old).

The institutional discourse, as well as the interviews with the founding partner, show a desire to do something for the local area, combined with a sense of opportunity. Xicos sought to respond to a local need by creating a network based on a web of relationships between organisations, people and companies, which, in addition to generating work and profit, also brings dynamism to the area, something that is clearly satisfying for the founders:

“It was always this idea: to deliver to people’s homes and give them this comfort, a service that was not yet available here in the area. So everything was in place for it to be a success.” (Founding partner, male, 25 years old).

In this context, we highlight the case of a client who says that because the organisation she works for has a lot of traffic in the restaurants area at lunchtime, it is common for them to place collective orders with Xicos: “We place collective orders (...) it’s more practical, we do not have to leave our workplace” (Client, female, 39 years old).

Xicos has tried to promote corporate social responsibility in a very simple way, by offering a daily discount to certain entities. During the Covid-19 pandemic, Xicos supplied clients working in essential services (security forces, health services) without charging any delivery fees. It has also worked with local municipalities in processes to promote support for vulnerable populations, with deliveries guaranteed by the municipality. A possible protocol with an NGO supporting the elderly population is currently being analysed.

## 4 Discussion and concluding remarks

The description of the results in the previous section highlights several characteristics that make Xicos’ mode of operation different from other platformed delivery companies and suggest new analytical approaches to the literature on platforms and the intensification of recent and alternative ones. Firstly, the profile of the company’s administration and its organisational operating dynamics tend to deviate from the trends observed in the platform economy (ILO, 2024). The fact that the company started with a clear start-up logic - an idea, little capital and people, with the partners taking on the role of couriers, and for a long time not having an app - was a hallmark of an organisational culture of experimentation that has a tradition in Portugal (Freire, 1995, Startup Startup Portugal, 2023). The young age of the founding partners and of the company itself may be a factor that reinforces this effect, which may eventually be lost as the company grows and with time and maturity. Nevertheless, the current organisational culture is characterised by a desire to innovate, to offer a service that combines the modern logic of platformisation with attention to the worker, the client and the promotion of the local area. The company’s external communication conveys this message very clearly. This finding is pertinent, as it challenges the approaches in scientific literature that highlight the narrative of a standardised and capital-oriented platformisation (Schor et al., 2020).

Another aspect in which Xicos seems to promote a different way of experiencing the reality of work and consumption in the context of platforms is by encouraging sociability through the strong intersubjective component that involves clients, couriers and other workers, as well as partner restaurants. With regard to couriers, Xicos shows a concern for organising work and managing people, without resorting to the algorithmic logic typical of platforms that offer tasks, define working hours, calculate payments, add evaluation mechanisms and even determine whether couriers remain connected to the platforms (ILO, 2024). Concerning clients, in contrast to the profile of a markedly solitary prosumer (Alonso and Fernández Rodríguez, 2021), the logics of engagement identified in the Xicos case study seem to create the possibility of proximity and collaborative relationships that maintain the strong physical and bodily component typical of client support services alongside the use of the platform. This service relation brings the consumer closer to an ‘electronic homo socius’, as opposed to the ‘electronic homo economicus’ suggested in the literature (Alonso and Fernández Rodríguez, 2021). With regard to the partner restaurants, there is also this logic of accompaniment and proximity, in the sense that when Xicos establishes these commercial partnerships, it favours a line of permanent dialogue, which is absolutely necessary since for many of these restaurants it is their first experience with the delivery system. In doing so, the company fulfils what could be called the external dimension of social responsibility (European Commission, 2001). In other words, corporate social responsibility goes beyond the sphere of the company itself and is extended to the local community involving business partners. This finding adds to the analytical approach to the literature on platforms considering that the platformisation appears to be a much more negotiable process than standardised work and consumption process (Ecke, 2022). However, it must be emphasised that Xicos’ growth logic to other medium size cities has the potential to distance it from the paradigm of functioning associated with its foundation.

In a place with a low population density and an increasingly ageing population, this profile of action gives Xicos a social innovation character, which takes on great importance, since it means that the company brings new ideas that contribute to solving urgent needs in the local area. Xicos is currently playing a central role in urban dynamics (Richardson, 2020), operationalising what can be called digital social innovation (Qureshi et al., 2021), as it involves the use of digital technologies in the development and implementation of innovative business models and services aimed at improving the well-being and action of socially vulnerable groups. The differentiated and so far very successful way in which Xicos combines tradition with technological innovation seems to be the key not only to its impressive growth (reaching a turnover of one million euros in 2023), but also to its validation as a benchmark in the Portuguese delivery market. This empirical research adds new insights to the literature on platforms as it underlines the extent to which urban ecosystems can be based on this profile of platformisation of delivery services.

The purpose of this article is to present and analyse the preliminary results of an ongoing investigation into the Xicos platform company. From what has been described and discussed above, it is clear that the strategic and operational management of the company currently differs from the dominant operation of the platform economy (ILO, 2024). The continuation of this empirical research will allow us to discuss the extent and direction in which this differentiated profile will change as the company grows, both in terms of the number of employees and partnerships, but also in terms of turnover growth, in the context of a foreseeable increase in competition.

## Data Availability

The raw data supporting the conclusions of this article will be made available by the authors, without undue reservation.

## Footnotes

^1^Traditional Portuguese dish.

## References

[ref1] Alonso L. E. Fernández Rodríguez C. J. (2021). El papel del consumo en la economía de plataformas: el vínculo oculto. Rev. Esp. Sociol. 30, 1–12. doi: 10.22325/fes/res.2021.69

[ref2] Aroles J. Mitev N. Vaujany F.-X. (2019). Mapping themes in the study of new work practices. New Technol. Work Employ. 34, 285–299. doi: 10.1111/ntwe.12146

[ref3] Cornelissen J. P. (2020). Corporate communication: A guide to theory and practice. Sage Publications.

[ref4] Cruz S. A. Gameiro A. (2023). Digital work platform: understanding platforms, workers, clients in a service relation. Front. Sociol. 7:1075808. doi: 10.3389/fsoc.2022.1075808 36687015 PMC9845709

[ref9001] Da Silva A. A. (2020). Teletrabalho, empresas-plataforma e o estatuto sociolaboral dos trabalhadores [Conference session]. Conferência Internacional sobre “As plataformas digitaise o futuro do trabalho”, online. Available at: https://colabor.pt/wp-content/uploads/2020/12/Ana-Alves-da-Silva.pdf

[ref6] Del Bono A. (2019). Trabajadores de plataformas digitales: Condiciones laborales en plataformas de reparto a domicilio en Argentina. Cuestiones de Sociología 21:83. doi: 10.24215/23468904e083

[ref7] Ecke Y. (2022). FairDelivery? Potential for and limits to alternative Platformization in Anke Strüver and Sybille Bauriedl (eds.) (2022) Platformization of Urban Life Towards a Technocapitalist Transformation of European Cities p.171. Bielefeld: transcript Verlag.

[ref9] European Commission (2001). Directorate-General for Employment, Social Affairs and Inclusion, promoting a European framework for corporate social responsibility - green paper. Publications Office.

[ref10] Freire J. (1995). Trabalho Independente em Portugal. Cies_Iscte. Lisboa: Centro de Investigação e Estudos de Sociologia (CIES).

[ref9002] Graham M. Woodcock J. Heeks R. Mungai P. Van Belle J. P. du Toit D.. (2020). The fairwork foundation: Strategies for improving platform work in a global context. Geoforum 112, 100–103. doi: 10.1016/j.geoforum.2020.01.023, PMID: 16408282

[ref90001] Heiland H. (2022). Neither timeless, nor placeless: Control of food delivery gig work via place-based working time regimes. Human Relations. 75, 1824–1848. doi: 10.1177/00187267211025283

[ref11] ILO (2018). Digital labour platforms and the future of work: towards decent work in the online world. Available at: https://www.ilo.org/global/ publications/books/WCMS_645337/lang--en/index.htm (Accessed May 6, 2024).

[ref12] ILO (2021). World employment and social outlook: the role of digital labour platforms in transforming the world of work. Available at: https://www.ilo. org/global/research/global-~reports/weso/2021/WCMS_771749/lang—en/index.htm (Accessed May 6, 2024).

[ref13] ILO (2024). Realizing decent work in the platform economy. Available at: https://www.ilo.org/resource/conference-paper/realizing-decent-work-platform-economy

[ref90002] Loureiro R. (2021). Restaurants adaptations in the online delivery channel during the Covid-19 pandemic. Lisboa: Nova School of Business and Economics.

[ref14] Machado D. D. P. N. Gomes G. Trentin G. N. S. Silva A. (2013). Cultura de inovação: elementos da cultura que facilitam a criação de um ambiente inovador. RAI Revista de Administração e Inovação 10, 164–182. doi: 10.5773/rai.v10i4.978

[ref15] Melewar T. C. Sarstedt M. Hallier C. (2012). Corporate identity, image and reputation management: a further analysis. Corp. Commun. Int. J. 17. doi: 10.1108/ccij.2012.16817aaa.002

[ref16] Mintzberg H. (2023). Understanding organizations…finally! Oakland: CA, Berrett-Koehler Publishers.

[ref17] Pesole A. Urzi Branca M. C. Fernandez Macias E. Biagi F. Gonzalez Vazquez I. (2018). Platform Workers in Europe Evidence from the COLLEEM survey. Luxembourg: Publications Office of the European Union.

[ref18] Qureshi I. Pan S. L. Zheng Y. (2021). Digital social innovation: an overview and research framework. Inf. Syst. J. 31, 647–671. doi: 10.1111/isj.12362

[ref5] Pereira C. (2024). Nova lei já levou a decisões para integrar dez estafetas. Jornal de Negócios, Emprego, Lei Laboral.

[ref9003] Ribeiro O. (1949). A Cova da Beira. Controvérsia de Geomorfologia”, Comunicações dos Serviços Geológicos de Portugal, Lisboa XXX, p. 4–23

[ref19] Richardson L. (2020). Coordinating the city: platforms as flexible spatial arrangements. Urban Geogr. 41, 458–461. doi: 10.1080/02723638.2020.1717027

[ref20] Sasikumar S. K. Sersia K. (2020). Digital platform economy: overview, emerging trends and policy perspectives. Productivity 61, 336–347. doi: 10.32381/PROD.2020.61.03.8

[ref21] Schor J. B. Attwood-Charles W. Cansoy M. Ladegaard I. Wengronowitz R. (2020). Dependence and precarity in the platform economy. Theor Soc 49, 833–861. doi: 10.1007/s11186-020-09408-y PMC7410973 32836676

[ref22] Startup Portugal (2023). Startup & Entrepreneurial Ecosystem Report, Portugal. IDC.

[ref23] Urde M. Greyser S. (2016). The corporate brand identity and reputation matrix – the case of the Nobel prize. J. Brand Manag. 23, 89–117. doi: 10.1057/bm.2015.49

[ref24] Vallas S. Schor J. B. (2020). What do platforms do? Understanding the gig economy. Ann. Rev. Sociol. 46, 273–294. doi: 10.1146/annurev-soc-121919-054857

[ref25] van Doorn N. Vijay D. (2021). Gig work as migrant work: the platformization of migration infrastructure. Environ. Plan. A Econ. Space 56, 1129–1149. doi: 10.1177/0308518X211065049

